# Beyond Colonoscopy: Exploring New Cell Surface Biomarkers for Detection of Early, Heterogenous Colorectal Lesions

**DOI:** 10.3389/fonc.2021.657701

**Published:** 2021-07-05

**Authors:** Saleh Ramezani, Arianna Parkhideh, Pratip K. Bhattacharya, Mary C. Farach-Carson, Daniel A. Harrington

**Affiliations:** ^1^ Department of Diagnostic and Biomedical Sciences, The University of Texas Health Science Center at Houston, School of Dentistry, Houston, TX, United States; ^2^ Department of Cancer Systems Imaging, The University of Texas MD Anderson Cancer Center, Houston, TX, United States; ^3^ MD Anderson Cancer Center UTHealth Graduate School of Biomedical Sciences, Houston, TX, United States; ^4^ Department of Anthropology, Washington University in St. Louis, St. Louis, MO, United States; ^5^ Departments of BioSciences and Bioengineering, Rice University, Houston, TX, United States

**Keywords:** colorectal cancer, biomarkers, colonoscopy, cell surface protein, adenocarcinoma, early detection

## Abstract

Colorectal cancer (CRC) is the third leading cause of cancer-related deaths among both men and women in the United States. Early detection and surgical removal of high-risk lesions in the colon can prevent disease from developing and spreading. Despite implementation of programs aimed at early detection, screening colonoscopies fail to detect a fraction of potentially aggressive colorectal lesions because of their location or nonobvious morphology. Optical colonoscopies, while highly effective, rely on direct visualization to detect changes on the surface mucosa that are consistent with dysplasia. Recent advances in endoscopy techniques and molecular imaging permit microscale visualization of the colonic mucosa. These technologies can be combined with various molecular probes that recognize and target heterogenous lesion surfaces to achieve early, real-time, and potentially non-invasive, detection of pre-cancerous lesions. The primary goal of this review is to contextualize existing and emergent CRC surface biomarkers and assess each’s potential as a candidate marker for early marker-based detection of CRC lesions. CRC markers that we include were stratified by the level of support gleaned from peer-reviewed publications, abstracts, and databases of both CRC and other cancers. The selected biomarkers, accessible on the cell surface and preferably on the luminal surface of the colon tissue, are organized into three categories: (1) established biomarkers (those with considerable data and high confidence), (2) emerging biomarkers (those with increasing research interest but with less supporting data), and (3) novel candidates (those with very recent data, and/or supportive evidence from other tissue systems). We also present an overview of recent advances in imaging techniques useful for visual detection of surface biomarkers, and discuss the ease with which these methods can be combined with microscopic visualization.

## Introduction

Colorectal cancer (CRC) remains the third leading cause of cancer-related deaths among both men and women in the United States, primarily because asymptomatic early disease often has progressed to an aggressive disease before it is diagnosed (1). Current American Medical Association (AMA) guidelines for early detection of CRC, adopted in 2018, recommend screening by fecal occult blood testing, sigmoidoscopy, or optical colonoscopy. In some health centers, CT colonography (“virtual colonoscopy”) also is available. Screening recommendations include starting at age 50, 45 for African Americans, and continuing until age 75 (2). As illustrated by the recent shocking death of actor Chadwick Boseman from colon cancer at age 43, early onset CRC increasingly is being diagnosed in younger patients who present with stage III/IV cancers (3). When diagnosed early, the 5-year survival rate of CRC is 90%, but the survival rate drops drastically with a later-stage diagnosis. According to the American Cancer Society (ACS), the 5-year survival rate of CRC for patients diagnosed at stage III and IV are 71% and 14%, respectively (1).

Several modalities are in current clinical practice for the screening of adenomatous polyps and CRC. Optical colonoscopy is the most widely used CRC screening technique, and the most commonly performed endoscopic procedure in the United States (4). Despite recent efforts to implement screening programs, full adherence to colonoscopy screening has proved elusive (5). It is estimated that 35% of adults in the United States who are eligible for colonoscopy screening remain unscreened (6). A survey of over 425 ethnically and racially diverse adults ≥ 50 years of age reported fear of embarrassment, fear of getting AIDS, fear of procedural pain, and older age as reasons to avoid colonoscopy (7). In addition, aversion to bowel preparation and fear of invasive procedures are among other barriers to undergoing a colonoscopy (8).

Alternative minimally invasive screening methods, such as fecal and blood tests, rely on changes in the tumor microenvironment to suggest the presence of CRC lesions, but these methods seldom detect early lesions in time to change the course of disease (9). While the new-generation immunochemical fecal occult blood tests (iFOBT) demonstrate a significantly higher diagnostic performance when compared with the earlier guaiac-based gFOBTs, a positive result still requires the use of a follow up colonoscopy (9).

CRC is a heterogeneous disease, arising from accumulations of various stochastic genetic and epigenetic alterations, which first lead to polyp growth and then, in some cases, to dysplasia and carcinogenesis. Polyps with similar morphological features typically follow different molecular paths to overgrowth. This observation is true even for lesions in hereditary forms of CRC such as those that occur in Lynch Syndrome (10). Because optical colonoscopies depend on the direct visualization of dysplastic tissue, lesions must reach a certain size and have an atypical appearance to be detected during the endoscopy procedure.

Sessile serrated adenomas/polyps (SSA/P), for instance, are usually flat, depressed, or similar in color to the surrounding healthy mucosa. These polyps can be under-recognized or mistaken for inverted colonic diverticulum (ICD) because of their shiny, smooth-surfaced appearance and a central indentation (11). It is estimated that among those who undergo optical colonoscopy, 3.5 per 1000 persons develop CRC adenomas that were missed during the baseline colonoscopy (12).

Missed adenomas during colonoscopies are relatively common: a meta-analysis of 43 publications and over 15,000 tandem colonoscopies estimated the miss rate to be 26%, 9%, 27%, and 34% for adenomas, advanced adenomas, serrated polyps, and flat adenomas, respectively (4). Although invasive carcinomas are present in only 5.4% of flat elevated lesions, 73.2% of flat depressed lesions between 10-20mm grow rapidly and become invasive carcinomas at an early stage (13). Given that the standard recommended screening interval is 10 years, a missed flat lesion could develop readily into advanced disease in the interval between serial colonoscopies (14). Given the high prevalence of CRC and the limitations of optical colonoscopy, the development of more sensitive, specific, and less invasive diagnostic techniques is warranted to identify “difficult to see” lesions and improve clinical outcomes for CRC patients.

### Biomarkers in Detection of Colorectal Lesions

Recent advances in molecular technologies have led to the discovery of multiple biomarkers that might facilitate early detection of colorectal lesions (15). The screening tests based on diagnostic biomarkers may be classified broadly as eliminating metabolites and circulating. Eliminating metabolite tests offer the opportunity to detect hemoglobin (through a peroxidase reaction), DNA or RNA, proteins such as M2 pyruvate kinase (M2-PK), and microbiome such as *Streptococcus bovis*, *Fusobacterium nucleatum* and *Helicobacter pylori* in stool (16). The circulating tests, used primarily as patient monitoring tools, are designed to identify CRC-specific biomarkers such as Carcinoembryonic Antigen (CEA) and carbohydrate antigen (CA19-9) in blood circulation (17).

Reasons for slow adoption of biomarker-based screening tests include the need for high throughput genotyping and phenotyping techniques, and challenges in achieving regulatory requirements for routine clinical use (18). The current biomarker screening tests require laboratory testing that is time-consuming and inconvenient. As histological analysis of biopsies remains the gold standard for a definitive CRC diagnosis (19), all abnormalities identified through biomarker tests next must be verified by colonoscopy during which removal of visible lesions may occur. To minimize the number of invasive procedures, it is typical for all suspicious lesions to be removed during the colonoscopy procedure. The fixation of the biopsy specimen, histological staining, and evaluation of the results complete the screening workflow.

**Figure 1:**
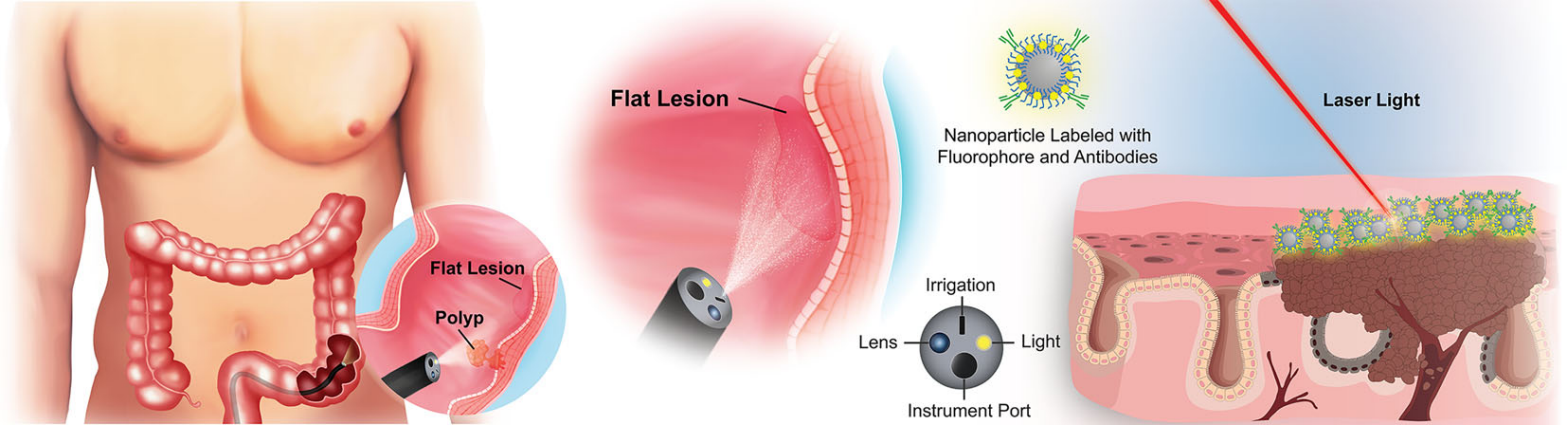
Overview of targeted imaging approaches to identifying flat colorectal lesions. For lesions that are not easily identified by traditional white light colonoscopy, a modified endoscope can both deliver high-contrast particles across tissue surfaces, and identify their retention through fluorescence barcoding, or *via* other sensitive visualization methods. Such “nanobeacons” could reveal otherwise undetectable early lesions.

The ideal biomarker(s) for early detection of pre-cancerous colorectal lesions should be highly sensitive and specific, expressed reliably on pre-cancerous or early stage lesions, and allow seamless integration into existing clinical protocols to eliminate unnecessary steps in the screening workflow (20). Because most colorectal lesions originate from the epithelial cells lining the luminal surface of the colon or their stem/progenitor cell precursors, biomarkers expressed on the polyp or lesion surface present an opportunity for early, real-time, *in vivo* detection of pre-cancerous lesions with high contrast against nearby healthy tissue (**Figure 1**). In an ideal world, a single perfect biomarker would instantly identify all lesions, regardless of phenotype. Unfortunately, the high degree of heterogeneity of colorectal polyps/lesions, both in stage and mutations, makes this notion unlikely. It is possible, however, to envision a minimum suite of biomarkers that would collectively identify the majority, if not all, of the lesions with a high risk of becoming cancerous. The necessary chemistries for tethering antibodies, aptamers, or other high-affinity recognition molecules to a contrast agent would still apply, regardless of the selected biomarker(s). With this motivation, we surveyed the literature to identify new potential early surface markers that could be combined with the established surface markers to create a future “visualization panel” that could be combined with colonoscopy or emerging methodologies to revolutionize CRC screening.

### Literature Review for Early Surface Biomarkers of CRC

**Figure 2:**
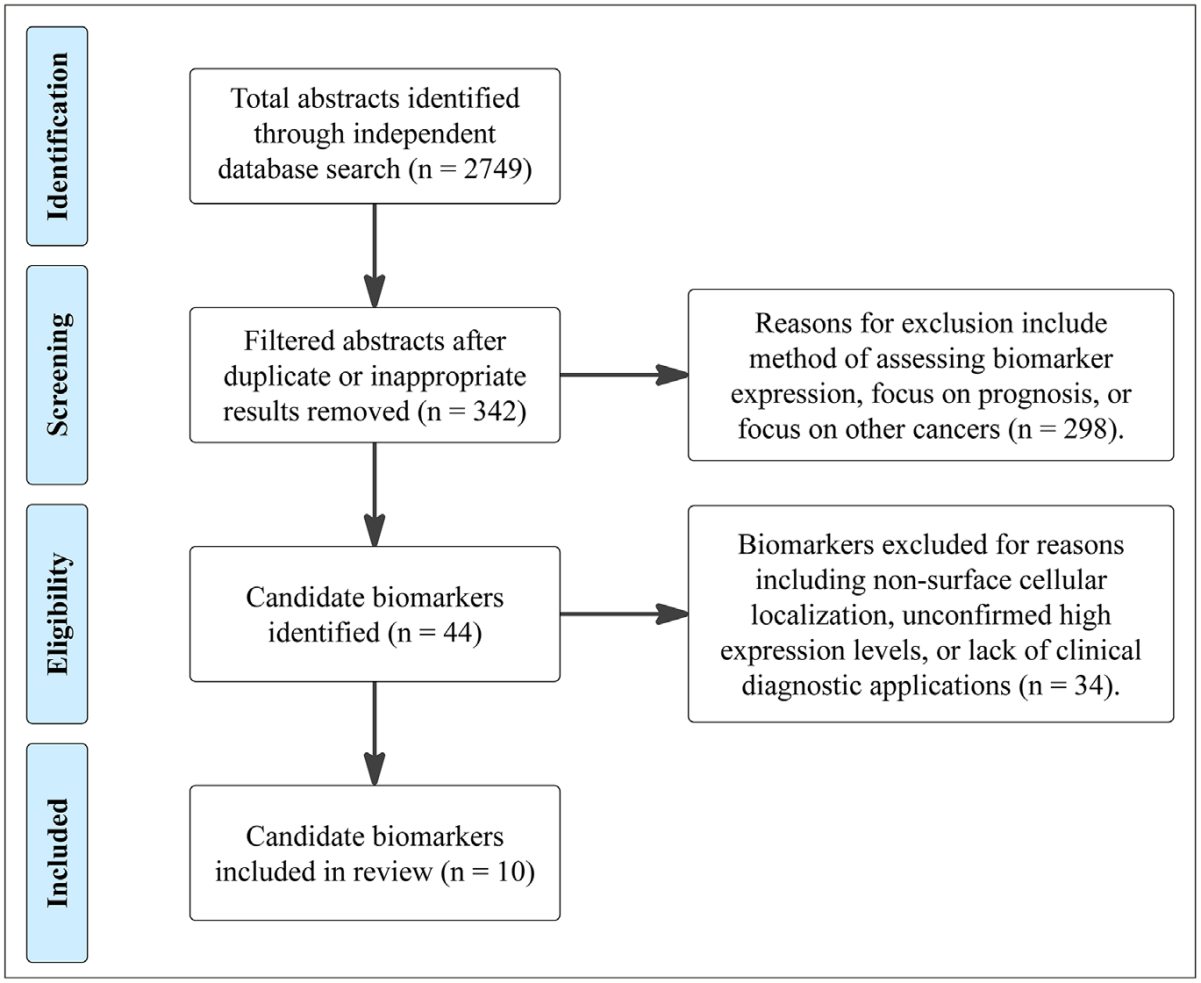
PRISMA diagram of selection criteria for biomarkers explored in this review.

We collected 2,749 abstracts relating to proteins or other cell-surface CRC biomarkers from a variety of databases using evolving search terms and their synonyms: “colorectal cancer”, “biomarker” “cell-surface protein”, “adenocarcinoma”, “EMT”, and “cancer stem cell”. These terms were searched in AACR abstracts, ASCO Meeting Library, Cancer Epidemiology, Biomarkers, Prevention, Cochrane Library, EBSCO Databases, Embase, and PubMed (**Figure 2**). With an intention of focusing on relatively new discoveries, we limited results to exclude studies published before the year 2000 but did not exclude based on method of evaluating protein expression, such that a diversity of methods and results could be considered for each biomarker. Furthermore, studies that illuminated new implications in oncogenic pathways or other systems of interest were reviewed. 342 biomarkers were selected and evaluated further. From these 342 biomarkers, 44 were identified based on biomarker expression and excluded those with focus on prognosis or on other cancers. These candidate biomarkers were further refined to 10 CRC biomarkers after excluding non-surface cellular biomarkers and those with unconfirmed high expression levels or lack of diagnostic applications.

## Surface Biomarkers

Recent studies on CRC biomarker discovery have provided excellent overview of diagnostic, predictive, and prognostic biomarkers identified through proteomics research (21), as well as those with therapy applications (22). Epidermal growth factor receptor (EGFR) (23), vascular endothelial growth factor (VEGF) (24), and mesenchymal-epithelial transition (c-MET or MET) (25) are among the well-established pathways that play an important role in the biology of CRC. A number of therapy agents targeting molecular biomarkers in these pathways, including *cetuximab*, *panitumumab*, and *bevacizumab*, have been developed and approved by the Food and Drug Administration (FDA) (26). However, these biomarkers are not currently being targeted clinically for early detection of CRC.

**Figure 3:**
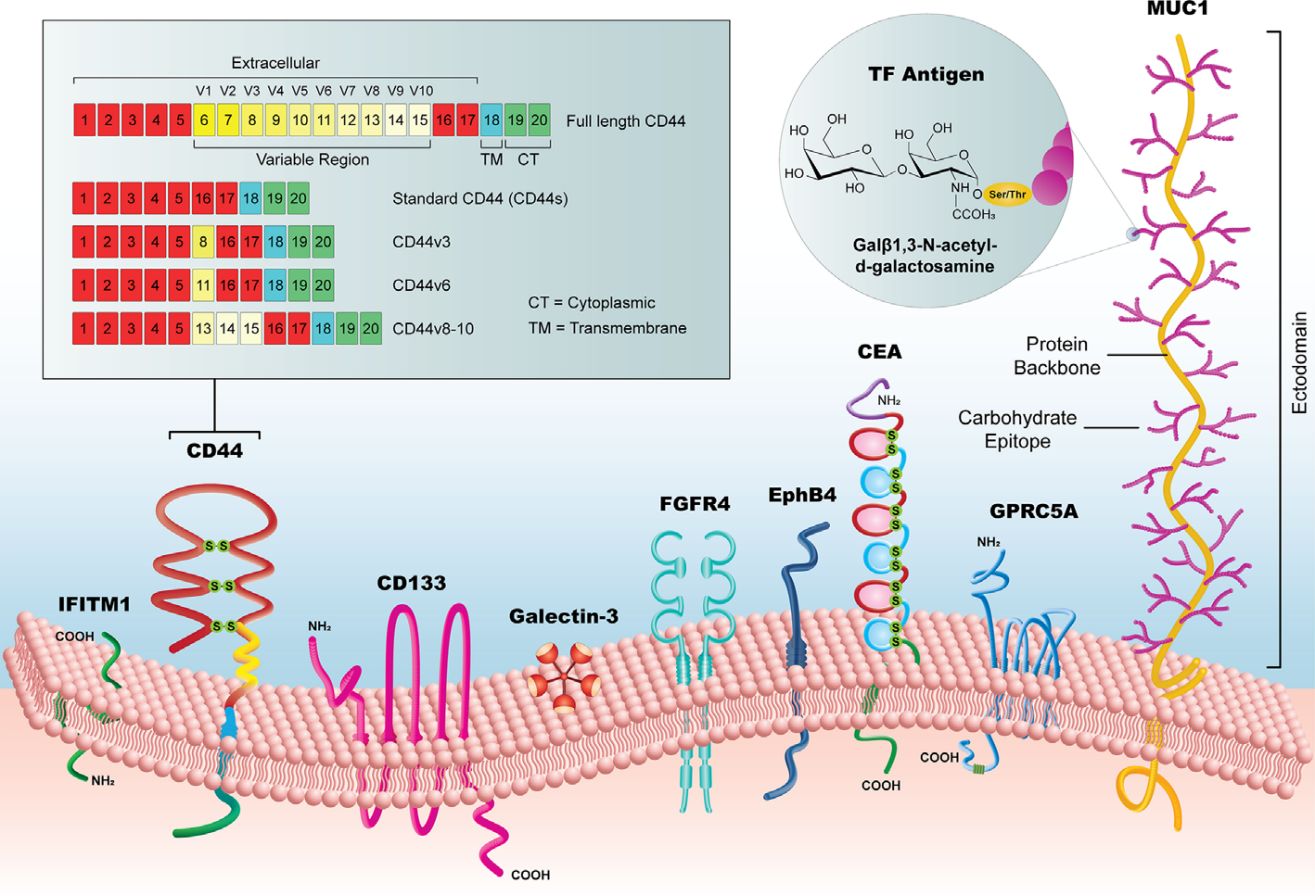
Summary of new candidate biomarkers, highlighted in this review. Although substantially variable in size, from the < 1kDa TF Antigen to the >1 MDa MUC1 glycoprotein, these surface biomarkers offer a potential to reveal early CRC lesion development, particularly when used as a library of markers that could identify lesions with heterogeneous subpopulations.

The selected biomarkers in this review are organized into three categories (1): established biomarkers, which have high supporting evidence in the literature and/or are currently in use in clinical practice (2), emerging biomarkers, which have had an increasing research focus and/or some evidence of utility in the pre-clinical literature, and (3) novel candidates, which have been recently implicated in CRC pathways or for CRC diagnosis, but for which there is limited pre-clinical or clinical evidence. It is important to note that the biomarkers listed in our review are not a comprehensive list but are representative of biomarkers identified by our selection criteria. We focused specifically on extracellular markers with sufficient surface accessibility in the colon to allow their identification by a functionalized probe. All biomarkers are listed in **Table 1**, with supporting literature evidence, and illustrated in **Figure 3** as a comparison of prominent molecular features and relative size.

**Table 1:** Surface biomarkers discussed in this review and the major features of included studies.

Surface Biomarker	Analysis	Stage	Tumor Location	Surface Expressed	Sample # (Ref)	Status
***Established Biomarkers***						
CEA	IHC, ISH	Dukes B, C	R, L, TV, C, S	Yes	16 (27)	FDA Approved^1^
CEA	IHC	I-IV	N/D	Yes	280 (28)	
CD133	IHC	I-IV	R, L	N/D	137 (29)	Clinical Trial^2^
CD133	IHC	I-IV	R, L, TV, RT, C, S	N/D	523 (30)	
CD133	IHC	-	R, L, TV, RT, C, S	Yes	200 (31)	
MUC1	IHC	-	R, L, TV, RT, C, S	N/D	45 (32)	Clinical Trial
MUC1	IHC	I-III	R, L, RT	Yes	381 (33)	
***Emerging Biomarkers***						
CD44s	IHC	I-IV	R, L, TV, RM	Yes	54 (34)	Clinical Trial
CD44s	IHC, PCR, ISH	-	N/D	Yes	10 (35)	
CD44s	IHC	I-IV	R, L, TV, RT, C, S	N/D	60 (36)	
CD44s	IHC	I-IV	R, L, RM	Yes	96 (37)	
CD44v3	IHC, PCR, WB	Dukes B, C, D	N/D	N/D	37 (38)	
CD44v6	IHC	I, III	N/D	Yes	234 (39)	
CD44v6	IHC	-	N/D	N/D	68 (40)	
LGALS3	IHC	Dukes A/B, C/D	Colon, RT	N/D	61 (41)	Clinical Trial
LGALS3	IHC, PCR, ISH	I-III	Colon, RT	N/D	57 (42)	
LGALS3	IHC, PCR	I-IV	R, L, TV, RT, C, S	N/D	201 (43)	
IFITM1	IHC, PCR, WB	I-IV	Colon, RT	N/D	229 (44)	Clinical Research
TF	IHC, PCR	-	Colon, RT	Yes	40 (45)	Clinical Research
TF	IHC	-	N/D	N/D	50 (46)	
***Novel Candidates***						
GPCR5a	IHC, WB	I-III	N/D	Yes	367 (47)	Clinical Research
EphB4	IHC	I-IV	R, L	N/D	168 (48)	Clinical Research
EphB4	IHC	I-IV	N/D	N/D	200 (49)	
FGFR4	IHC	I-III	N/D	N/D	43 (50)	Clinical Trial

^1^List of Cleared or Approved Companion Diagnostic Devices (www.fda.gov).

^2^NIH U.S. National Library of Medicine Clinical Trials (www.clinicaltrials.gov). Biomarkers are categorized as those 1) having a strong consistent presence in the literature (established), having an increasing focus in the literature (emerging), and those that have been implicated recently in CRC pathways or for CRC diagnosis (novel). Surface expression is defined as detectable in the luminal surface of the colon. IHC, immunohistochemistry; PCR, real time PCR; ISH, In situ hybridization; IF, immunofluorescence; WB, western blot; R, Right; TV, Transverse; RT, Rectum; C, Cecum; S, Sigmoid; N/D, Not Determined; Dukes A: invasion into but not through the colorectal wall; Dukes B: invasion extends through muscularis or invades adjacent organs (no lymph node involvement), Dukes C: invasion involves lymph nodes, Dukes D, invasion involves distant metastasis.

### Established Biomarkers

#### CEA

Carcinoembryonic antigen (CEA) is a glycoprotein that has been implicated as a CRC biomarker since 1965. It is a member of the CEACAM family, which contains 12 independent genes. CEA, or CEACAM5, is associated with the cell membrane through a glycosylphosphatidylinositol (GPI) anchor (51). CEA is involved in mediating homophilic and heterophilic interactions, and has an understood role in cell-cell adhesion, inter and intracellular signaling, vascularization, and a variety of other physiological functions (51, 52).

CEA is expressed in healthy fetal and adult tissue in various organ systems, including the digestive tract (53). A 1993 study found that both mRNA and tissue-level expression of CEA in paired healthy and cancerous resected colon samples had a similar expression gradient in colonic crypts. The maximum amount of CEA expression was in the upper third of the colonic crypt, with a gradient of decreasing amount towards the base (27). While CEA is expressed in healthy tissue, overexpression of CEA in a variety of cancers, including breast, gastrointestinal, and respiratory, has had a role in diagnosis and prognosis since its discovery (52).

Immunohistochemical studies have highlighted the role that CEA can play as a diagnostic biomarker in CRC. Matched FFPE samples of normal colorectal mucosa and primary carcinoma stained for various biomarkers found the greatest difference in degree of expression for CEA (28). This study determined that the sensitivity and specificity of CEA used for CRC imaging would be 93.7% and 96.1% respectively. Because of this, CEA-detecting diagnostic methods have been developed and used on mouse models with human breast and pancreatic cancer and have been successful in detecting primary tumors and micrometastases (54).

The utility of serum CEA levels as a prognostic marker was demonstrated multiple times in the past 20 years (55, 56). Elevated CEA levels in serum was linked to poor prognosis in CRC patients, and was shown to independently predict higher overall mortality in both metastatic and nonmetastatic CRC patients, and across all cancer stages (57). Notably, elevated CEA is also observed in IBD and other inflammatory diseases, reducing its utility as a single marker for early CRC. Its use in combination with other candidates in this review may enhance its specificity.

#### CD133

CD133, also known as Prominin-1 or AC133, is a pentaspan transmembrane glycoprotein protein that often is recognized as a stem cell marker for normal and caner tissue (58). Encoded by gene PROM1, CD133 has some cell-cycle dependence, and its expression may be promoted in hypoxic environments, a key characteristic of the tumor microenvironment and of the colon (59, 60). CD133 is linked to tumor-forming and tumor-growing related processes, and is associated with an increase in tumor volume and tumorigenicity (61).

Although CD133 often is cited as a cancer stem cell marker (59), its role as a stem cell biomarker was disputed in one study. Yeung and Mortensen found that only one in 262 CD133+ cells can initiate tumors (62). Others question the efficacy of CD133 as an EMT marker as well, as it was found to localize to the nucleus during EMT, and return to the cell membrane when MET is induced (63). These findings indicate that cell-surface expression of CD133 is more likely in the early stages of CRC, which makes it a more useful tool for CRC screening.

Tissue expression of CD133 in CRC, though not without controversy, makes a strong case for its use as a CRC biomarker. Immunohistochemistry (IHC) staining of colon cancer tissue removed from a cohort of 137 patients showed high CD133 expression in 64% of stage I, 28% of stage II, and 54% of stage III tumors (29). More modest IHC results set the CD133 positive staining at 24.5% (30). However, CD133 presence in the healthy colon epithelium is controversial: Shmelkov et al. found that while CD133 is expressed ubiquitously in the majority of the human colon cancer cells, the healthy human colon also expresses CD133 robustly (58). It is worth noting that because of the glycosylation pattern of CD133, the choice of antibodies may impact the detection of this biomarker in colorectal tissue and cell line (64). Nonetheless, a metanalysis by Abbasian et al. showed higher levels of CD133 in cells derived from primary tumors than cells originated from cell lines (61).

While the relationship between CD133 expression and colorectal tumor volume or stage varies among studies (30, 31, 65), a meta-analysis of 27 tumorgenicity studies found a twofold increase in tumor volume in cells that expressed CD133 or CD44. Interestingly, co-expression of CD133 and CD44 was associated with sevenfold higher tumorgenicity (61), further implicating their combined application as surface biomarkers for *in vivo* screening of CRC.

#### MUC1

The non-neoplastic (normal) surface of the colon is lined with various types of mucins, secreted by specialized epithelial cells that protect the lining of the epithelium against invading bacteria and pathogens (66). In developing neoplasms, however, the mucin layer is decreased, presenting an opportunity for detection of surface accessible biomarkers (67). MUC1 is a large, highly accessible, transmembrane mucin that is expressed on the apical surface of many luminal epithelial cells of the respiratory, urinary, gastrointestinal, and reproductive tract (68). The mature glycosylated form of MUC1 has an estimated weight of 250-500kDa. In most epithelial cancers, including CRC, MUC1 is overexpressed and displays aberrant glycosylation (69). Thus, the glycosylated ectodomain of MUC1 can serve as a potential surface biomarker for *in-vivo* screening of CRC.

In one study, the immunohistochemical analysis of CRC tissues from 45 patients revealed positive expression of MUC1 in 55.6% of CRC tissue and in 0% of nontumor tissue adjacent to carcinoma (32). In another histopathological study of tissues removed from 381 CRC patients, it was discovered that MUC1 is expressed in 64% of the CRC tissue (33). The high expression of mature MUC1 mucin correlates with TNM stage, depth of invasion, lymph node metastasis, and poor overall survival (OS) outcome in CRC patients (70). These findings suggest that MUC1 is a promising prognostic and diagnostic surface biomarker of CRC.

### Emerging Biomarkers

#### CD44 Variant Isoforms

CD44 is a 85-200 kDa transmembrane glycoprotein that has various functions in cell division, migration, adhesion, and signaling (71–75). The broad variation in molecular weights is partially attributable to multiple aberrant splice variants, which may include several exons, and multiple variations in glycosylation state (76). The standard form of CD44 (CD44s) is made of common exons 1-5 and 16-20 that when spliced together form a transcript that encodes the isoform with the smallest molecular weight (85-90 kDa). The other 10 variable exons (6–15), also known as v1-v10, are excluded either completely from CD44s or are spliced and inserted between exons 5 and 16. Both the standard form and the higher-molecular weight isoforms (CD44v) of CD44 are expressed on the cell membrane, strengthening their status as potential surface biomarkers for early visual detection of CRC.

CD44s, the most abundant form of CD44, plays an important role in cellular adhesion and three dimensional organ and tissue maintenance (77). Because of its important role in cell-to-cell adhesion, CD44s dysregulation is implicated in multiple cancers, including breast, prostate, pancreatic, and colon cancer (78). Colorectal tissue expression levels of CD44s vary notably among studies. Several studies have reported increased expression of CD44s in CRC tissue compared to adjacent normal tissue (35, 79), with CD44s expression localized at the base of colonic crypts in a healthy colon and on the luminal side of crypt in CRC tissue (34, 35). Others have shown loss or down-regulation of CD44s during transformation from normal mucosa to colon carcinoma (80). The inconsistent expression levels of CD44s in colorectal adenomas, combined with weak to moderate expression in normal colon epithelium, undermine the efficacy of CD44s as a diagnostic biomarker for CRC.

CD44v6, another splice variant of CD44 containing exon v6, has a higher affinity for hyaluronic acid than CD44s, further implicating this CD44 variant in tumor pathology (81). The immunohistochemical staining of normal (n=25) and hyperplastic colon tissue (n=45) has shown that the expression of CD44v6 in the healthy colon is rare and limited to the base of colonic crypts (39). This is consistent with the finding that CD44v6 is found in only one out of 23 benign serous effusions from adenocarcinomas (39). In normal and hyperplastic colon tissue, CD44v6 expression was absent or sporadic in the crypts or lumen epithelium, but showed as strong/diffuse staining in the dysplastic surface epithelium of tubular adenomas (86%, 49/57), tubulovillous adenomas (84%, 21/25), and villous adenomas (100%, 9/9) (39). The low expression of CD44v6 in the healthy colon and its increased expression in the dysplastic surface epithelium in adenomas makes this biomarker a strong candidate for visual CRC screening technologies.

CD44v3 is the heparan sulfate proteoglycan domain of CD44 containing the sequence encoded by variant exon 3 (82). This isoform is thought to contribute to malignant behavior in human colon through reduced binding affinity for heparan sulfate, a molecule that enhances the invasive capacity of colon cancer cells (38). Immunohistochemical staining of normal colon mucosa and primary colon adenocarcinoma samples has shown significantly higher levels of CD44v3 in the cytoplasmic membrane of cancer cells compared to normal mucosal cells (38). CD44v3,8-10, an isoform variant containing both exons v3 and v8-10, is another high molecular-weight (~260 kDa) isoform of CD44 that could be relevant for diagnosis of CRC (83). The reverse transcriptase polymerase chain reaction (RT-PCR) analysis of transcripts present in mucosal samples, removed from 53 patients, detected CD44v3,8-10 transcript in 2/23 (6%) of normal, 19/20 (95%) of adenoma, and 29/31 (93.5%) of carcinoma (84).

The limited expression of CD44v3, CD44v6, and CD44v3,v8-10 splice variants in healthy colon tissue and their apparent upregulation in CRC tissue strengthens the potential use of these biomarkers for future CRC screening.

#### Galectin 3

Galectin 3, or LGALS3, is a member of the galectin family, a group of carbohydrate-binding lectins characterized by their binding affinity for beta-galactosides (85). LGALS3 is expressed at the cell surface, where it interacts with the extracellular matrix, especially with glycoproteins, and has the ability to affect intracellular signaling pathways (42). LGALS3-expressing cells also possess higher ALDH1 activity, which often correlates with a dedifferentiated cancer stem cell phenotype, than do their LGALS3-negative counterparts (86).

The correlation of LGALS3 expression in CRC with clinical pathological characteristics has been explored in several immunohistochemical and RT-PCR studies. In one study, the IHC staining of CRC tissue (n=61) and normal adjacent tissue (n=23) samples showed significantly higher LGALS3 expression in cancer tissue (62.5%) versus normal cancer-adjacent tissue (13.0%) (41). In another study, 75% of CRC tissue samples stain high for LGALS3, and ten CRC cell lines were shown to have increased LGALS3 protein levels compared to HeLa cells (42).

LGALS3 expression varies according to cancer staging and the degree of differentiation of the adenocarcinoma. LGALS3 mRNA levels were higher in early stage colorectal cancers (58% in stage I) compared to advanced cancers (50% in stage IV) (43). Protein analysis found higher LGALS3 levels in primary adenocarcinomas than in metastatic adenocarcinomas, and stronger LGALS3 staining in well-differentiated tumor areas compared to poorly differentiated tumor areas (43). Conversely, colorectal adenocarcinomas may display higher levels of LGALS3 than do colorectal adenomas; one study sets the rate of colorectal adenocarcinoma expression of LGALS3 at 95% while only 73% of adenomas were positive for LGALS3 (43). The higher expression of LGALS3 in CRC tissue compared to normal adjacent tissue, and high expressions in early stage CRC, make this biomarker a potential candidate for early diagnostic applications.

#### IFITM1

Interferon-inducible transmembrane protein 1 (IFITM1) is a member of the IFN-inducible transmembrane protein family that mediates the antiproliferative effects of cytokines. A 2008 meta-analysis of CRC gene expression profiles identified IFITM1 as consistently upregulated in cancer conditions *vs* normal tissue (87), building on prior evidence from *in situ* hybridization studies (88). Later staining by IHC of IFITM1 protein in tissue specimens confirmed elevated IFITM1 levels in CRC, compared to paired adjacent normal tissues (44). Oligonucleotide microarray comparisons between healthy tissue and tumor tissue also showed a 1.5-fold plus increase in IFITM1 levels in CRC (89). High levels of IFITM1 are positively correlated with distant metastasis, advanced stage, and poor OS (44).

Transfection of LoVo and HT-29 colorectal cancer cells with small interfering RNA (siRNA) targeting IFITM1 reduced migration and invasion capacities of these cells in a wound-healing assay, whereas overexpression of IFITM1 enhanced these capacities (44). In a separate study, IFITM1 overexpression in SW480 cells also promoted invasiveness (90). Conversely, IFITM1 reduction by >85% using short hairpin RNA (shRNA) reduced cell proliferation by 10-35% (91). Immunoblot analysis shows that non-metastatic SW480 had low levels of IFITM1, but metastatic cell lines such as HT29, HCT116, and SW620 exhibited higher levels of IFITM1 (91). Western blot conducted by another team found similar results: high IFITM1 levels in HCT116, LoVo, and HT-29 cell lines (44).

Whether in native tissue or in cell lines, this recent data all supports the notion that high levels of IFITM1 correlates with increased migration, invasion, and behaviors associated with metastasis in CRC.

#### Thomsen-Friedenreich Antigen

The Thomsen-Friedenreich (TF) antigen (also known as T antigen or CD176) is a disaccharide with the structure galactose β1,3-*N*-acetyl-d-galactosamine (Gal-GalNAc). Over many decades, TF antigen has been appreciated increasingly for its appearance on aberrantly glycosylated proteins, particularly cell surface proteins on transformed epithelia (92). More recent literature identified TF antigen in numerous cancer types, including CRC, as well as an interesting potential interaction of this uncommon disaccharide with the galectins (93). Because this antigen also is displayed on gut bacteria, anti-TF antibodies are found in humans, and are postulated to be agents for immunosurveillance of developing tumors (94).

The immunodiagnostic potential of TF antigen in patients with CRC has been investigated in several studies using IHC. In most normal mucosa, the TF structure is not present and is instead modified (45). In two separate immunohistochemical studies, the TF antigen was undetectable in normal colon mucosal tissue (46, 95), but was reported in 60% of adenomas and adenocarcinomas (46). These findings explain the rationale for exploiting the specificity and sensitivity of using the TF antigen as a nanobeacon target in CRC imaging techniques, such as shown in **Figure 1**.

The TF antigen has been the subject of a study of its potential use as a molecular target of targeted nanobeacons for fluorescent light (FL) colonoscopy (96). This study showed that in a xenograft CRC mouse model the fluorescent specimens had a 35-fold stronger signal than controls, which grew to 60-fold at a later stage of tumor development. The strong signal-to-noise ratio achieved when using the TF antigen as a target in imaging techniques is congruent with protein expression levels identified in other studies.

The TF antigen can also be detected using peanut agglutinin and other lectins, which can be more cost-effective than antibodies for functionalizing micro- or nano-particles (96, 97). The TF antigen is an exciting target to consider for future CRC imaging techniques as it has already been applied in this setting with promising results. This antigen can potentially reduce the costs associated with purchasing antibodies for functionalization.

### Novel Candidates

#### GPRC5A

GPRC5A is a retinoic acid induced G protein-coupled receptor (GPCR), class C, group 5, also known as retinoic acid-induced gene 3 (RAI3) or retinoic acid-induced gene 1 (RAIG1). The GPCRs are a broad family of transmembrane receptors, with a broad array of functions. GPRC5A is one of a group of four proteins within group 5, which is identified by the sequence similarity of its members (GPRC5A, -B, -C. and -D), and characterized by a short extracellular N-terminal domain, and its ability to be induced for transcription by retinoic acid (for all members except GPRC5D) (98). In normal tissues, GPRC5A is primarily expressed in lung, albeit followed next by the gastrointestinal system, including colon (99). Despite this, baseline GPRC5A transcripts are relatively low in normal adult colon tissue (100), and protein staining by IHC in normal colon tissue remains undetectable or limited to a few neuroendocrine cells within the colonic crypts (47).

Over the last 10-15 years, dysregulation of GPRC5A was identified in many cancers, including breast, prostate, and CRC (47, 98, 99). In one immunohistochemical study, staining of 367 CRC tumor samples displayed GPRC5A localization to the luminal membrane of 193 (62%) samples (47). In another study, the transcript levels were 2.2-fold higher in cancerous tissue than in healthy tissue of 57 paired patients (101).

The relationship between GPRC5A expression and prognosis are unclear. GPRC5A expression may be a negative prognosis factor, as an analysis of Gene Expression Omnibus databases found (101). GPRC5A expression may also be related to hypoxia, a key characteristic of many tumor microenvironments. Hypoxia-induced increases in the levels of GPRC5A was found in a stable isotope labeling by amino acids in cell culture (SILAC)-based proteomics analysis of SW640 cells grown in normoxia and hypoxia. GPRC5A deletion increased apoptosis in hypoxic conditions by 12.2-fold (102). These findings highlight the role of GPRC5A presence in CRC pathology, and strengthen its potential as a CRC biomarker.

#### EphB4

Ephrin type-B receptor 4 (EphB4) is a tyrosine kinase-type receptor that recognizes membrane-bound ephrin ligands on adjacent cells. One important role of ephrins and their receptors is to direct cell-cell positioning, through their bidirectional signaling and activation (103). These proteins are integral partners in the development of neural and vascular structures, and in the maintenance of tissue boundaries. Conversely, release of boundaries enables de-differentiated stem/progenitor cell phenotypes, and their dysregulation in cancer further permits the disruption of stable tissue boundaries, and ultimately favors a migratory and metastatic phenotype.

EphB4 expression in the healthy colon was reported as minimal to none (104, 105). However, EphB4 is overexpressed in multiple CRC cell lines, including SW480, LIM2405 B4, and CT26 cells, highlighting its potential as a CRC biomarker (106). Differences in IHC staining for EphB4 in tumor tissue and normal tissue was very pronounced according to one team (48). IHC staining of 50 normal colon tissue samples showed EphB4 levels high in only 8% of healthy samples (49), while others showed EphB4 expression in 73% and 85.3% of clinical CRC samples (104). EphB4 shows potential as a candidate biomarker because of the strong differences in its expression levels between healthy and cancerous tissue, and its implication in multiple processes related to cancer progression.

#### FGFR4

Fibroblast growth factor receptor four (FGFR4), one of the four transmembrane tyrosine kinase receptors, plays a role in cell proliferation, differentiation, and survival (107). Recent IHC studies found positive FGFR4 stains in 90.7% of biopsies removed from patients with locally advanced CRC (50). Protein levels of FGFR4 were higher in colorectal adenoma tissue as well (108).

FGFR4 levels may be related to metastasis and EMT. FGFR4 knockdown facilitated the expression of E-cadherin and decreased levels of TWIST and other EMT inducers (109). This data aligns with findings that metastatic CRC cell lines have higher FGFR4 levels than non-metastatic cell lines, but contrasts with the finding that FGFR4 is cancer-specific in early Duke’s stages (110). FGFR4 is a target of FOXC1, which when elevated is associated with worse prognosis, as is FGFR4 (111).

Although measures of FGFR4 levels in CRC through IHC remains limited, its elevation in other cancers and early evaluation of its protein levels at the cell surface suggests that FGFR4 should continue to be assessed as a CRC biomarker. The relationship between FGFR4 expression and cellular adhesion, invasion, and metastasis pathways highlights its potential as a CRC marker.

### Other Biomarkers With Specificity for Flat Lesions

Non-polypoid colorectal neoplasm (NP-CRN) represent a heterogeneous group of lesions that appear to be slightly elevated, completely flat, or slightly depressed compared to normal adjacent mucosa (112). SSA/P and hyperplastic polyps, both classified as NP-CRN, share some of the same histological and molecular features. Histologically, SSA/P and hyperplastic polyps both have crypts with serrated luminal outline and epithelial cells that are rich in mucin (113). Molecularly, both polyp subtypes have the BRAF mutation, a downstream target in the EGFR signaling pathway (114). The SSA/P, differ from hyperplastic polyps in their higher degree of abnormal proliferation and potential to develop invasive adenocarcinomas (115). Because histopathological classification of polyps is critical for determining the malignant potential of colorectal lesions, surface biomarkers that can differentiate between SSA/P and hyperplastic polyps would be highly valuable in accurate characterization of lesions.

#### Annexin A10

Although not highlighted in our larger group of biomarkers in **Figure 3**, Annexin A10 was found in our broader category of 44 candidate biomarkers (**Figure 2**), and has particular relevance here in relation to SSA/P lesions. Annexins (ANX) are a large family of eukaryotic calcium-dependent membrane proteins that play important roles in cell life cycle, exocytosis, and apoptosis (116). Some annexins (A1, A2, A4, A10, and A11) are expressed at higher levels in CRC than in normal colon (117, 118). Several studies showed that A10 (ANXA10) is correlated with serrated pathway of colorectal carcinoma (113, 119–121). A microarray analysis of distal hyperplastic polyps (n=6) and proximal SSA/Ps (n=6) showed that ANXA10 has a 73% sensitivity and 73% specificity in the diagnosis of SSA/Ps (113). In another immunohistochemical study with larger samples (n=131), immunoreactivity for ANXA10 predicted serrated histology with sensitivity of 55% and specificity of 97% (120).

#### CD133

CD133 was already noted in 2.1.2. as a relevant biomarker for early CRC. While not without controversy as a diagnostic and prognostic biomarker, the stem cell biomarker CD133 has been shown additionally to be implicated in the serrated pathway. In one immunohistochemical study, CD133 was expressed more prominently in SSA/P than in hyperplastic polyps (122). This finding further strengthens the status of CD133 as a potential biomarker for early detection of CRC.

## Imaging of Surface Biomarkers

**Figure 4:**
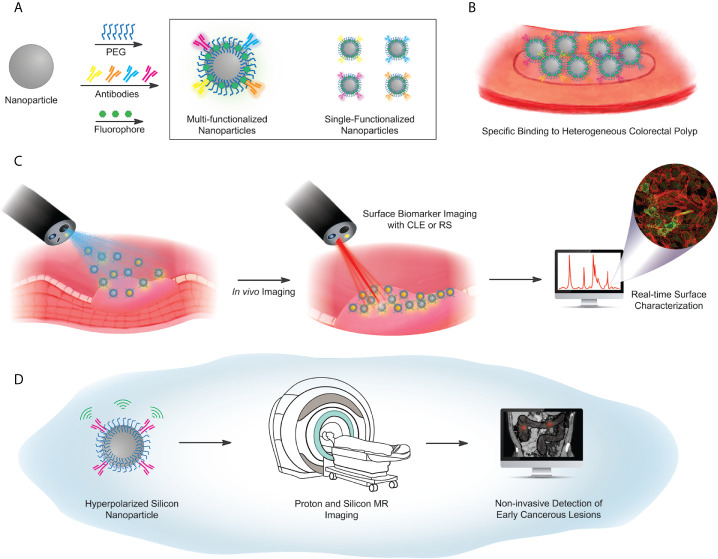
Proposed workflow for future concepts in particle-based targeted imaging. **(A)** Surface functionalization scheme, using a library of antibodies against selected surface biomarker targets. Antibodies are covalently coupled to a PEG-functionalized particle, with either a fluorescent barcode, or hyperpolarization (used in **(D)** below). Particle libraries may have multiple targeting antibodies on a single particle (box, left) or single-target particles, mixed to illuminate multiple biomarkers on a heterogeneous lesion (box, right). **(B)** Illustrated identification of lesions, *via* a library of particles. **(C)** Imaging workflow for fluorescence-based or Raman-based image capture. **(D)** Hyperpolarized Si particles preserve signal over a sufficient lifetime to enable magnetic resonance-based imaging. The MRI image of the abdomen adopted from the depiction of volvulus on Wikipedia under the Creative Commons license: https://en.wikipedia.org/wiki/Volvulus).

The key value to identifying a battery of cell surface biomarkers is that antibodies or other targeting agents for each biomarker can be tethered to various molecular contrast agents, with the aim to improve early identification of CRC that may be difficult to visualize by other means. In addition to visual detection of surface biomarkers, contrast agents can act as carriers for selective release of drugs in cancer cells (123). Existing imaging technologies such as magnetic resonance imaging (MRI), positron emission tomography (PET), confocal laser endomicroscopy (CLE), and Raman spectroscopy offer potential improvements to conventional colonoscopy methods (**Figure 4**).

### Confocal Laser Endomicroscopy

Confocal laser endomicroscopy (CLE) is an endoscopic modality that was developed for real-time histological assessment of the mucosal layer of the gastrointestinal (GI) tract (124) through the excitation and imaging of fluorescent probes that highlight specific tissue features. CLE has been used in the diagnosis and management of various diseases including squamous cell cancer of the esophagus, gastric cancer, gastritis, celiac disease, and to visualize colonic neoplasia and normal colonic mucosa (125–128). In the lower GI tract studies with CLE, systemic fluorescein combined with topical acriflavine have been used to distinguish high grade and low grade adenomas (129). These studies indicate that CLE can provide real-time histological information and aid in characterization and *in vivo* diagnosis of colonic lesions. Additionally, CLE can be combined with exogenous probes conjugated with fluorescein to detect tumor-specific biomarkers in CRC patients. In one study, fluorescein-conjugated peptides administered topically to dysplastic colon were detected using CLE with 81% sensitivity and 82% specificity (20).

### Raman Spectroscopy and SERS

Raman spectroscopy (RS) is another modality that has shown potential for endoscopic diagnosis of diseases of the epithelium, including those of the esophagus, stomach, and colon (130–132). RS can be combined with exogenous contrast agents, such as surface-enhanced Raman-scattering (SERS) nanoparticles (NPs), for sensitive and multiplexed molecular imaging of epithelial biomarkers. Several studies have reported the detection of targeted and non-targeted SERPS NPs in small animals and on human tissue (133–136). SERS NPs can be conjugated to antibodies, peptides, lectins or other moieties that target a diverse panel of tumor-enhanced biomarkers. Although SERS NPs have low toxicity, their application in clinical studies requires further regulatory approval. Nonetheless, Zavaleta et al. have developed and tested the utility of a Raman endoscope for detection of functionalized-SERS NPs in human patients. This Raman endoscope was inserted through the accessory channel of a conventional endoscope and acquired images from the colon wall of a human patient (137).

### Hyperpolarized MRI

In recent years, the implementation of virtual colonoscopy (CT and MRI) has emerged as potential alternatives to colonoscopy. However, these modalities historically suffer from significant drawbacks including poor detection accuracy for small (<10mm) lesions (138, 139), reliance on non-tumor specific contrast agents, and inaccurate diagnosis due to false-positives or false-negatives (140). One novel approach to increase the sensitivity of MRI is through dynamic nuclear polarization (DNP), whereby the nuclear spin alignment of underlying material is enhanced by 3-4 orders of magnitude, resulting in higher signal to noise ratio (SNR) (141). Hyperpolarized silicon (^29^Si) particles (HP SiPs), detectable *via* MRI, could serve as nanobeacons to discern cancerous tissue from healthy tissue in a variety of cancers including prostate, ovarian, and colorectal cancer (142–144).

Silicon particles can be functionalized with antibodies or other targeting moieties to detect specific cell surface biomarkers common to CRC, but largely absent from healthy tissue. The feasibility of imaging surface biomarkers *in vivo* with antibody functionalized HP SiPs has been demonstrated in a CRC mouse model expressing MUC1. 2μm HP SiPs functionalized with an IgG1 antibody to MUC1 (214D4), administered to human-MUC1 expressing mice (MUC1^+^) *via* the rectum, were detected at the location of the tumor. The ^29^Si MRI signal was absent in a control study where the same particles were administered to MUC1 negative mice (MUC1-), or in another control where PEGylated only particles (without the 214D4 antibody) were administered to human MUC1 expressing mice (MUC1^+^) (144).

### PET

[^18^F]Fluorodeoxyglucose PET has been clinically used for the evaluation of patients with a wide variety of cancers since most malignancies, including colorectal cancer, typically show increased glucose metabolism (145, 146). However the versatility of this receptor imaging technique can be harnessed (147–150), much like hyperpolarized Silicon-based MRI to functionalized appropriate biomarker antibodies/peptides/aptamers/affibodies for colorectal cancer with a radioactive nuclei (^18^F, ^68^Ga, ^124^I, ^89^Zr) to achieve targeted molecular imaging in colorectal cancer systems.

## Conclusions and the Future of Biomarkers in Cancer Screening

Although optical colonoscopy is the gold standard in CRC screening, its effectiveness in detecting early lesions critically depends upon the experience of the endoscopist and the ability to visually distinguish the lesion from normal tissue. To be visible through conventional endoscopes, colorectal lesions must reach a certain size and have an atypical appearance. This lack of specificity can lead to under-detection of potentially aggressive early lesions such as sessile serrated adenomas/polyps.

Because CRC almost always originates from the epithelial cells lining the luminal surface of the colon, surface accessible biomarkers present an opportunity for early detection of CRC. This means that instead of relying on direct visual detection of dysplastic tissue, one can use sophisticated imaging methods to search for cellular signatures that identify malignant or pre-malignant cells and growths. In this review, we surveyed the literature to identify potential surface biomarkers that might facilitate early detection of colorectal lesions. These biomarkers may be combined with colonoscopy or emerging methodologies to enhance the detection of heterogeneous tumors in diverse patient populations. The objective of future research will be to explore a cost-effective and non-invasive approach for early and real-time detection of pre-cancerous lesions with high contrast against nearby healthy tissue. Other goals are to create unique spectral signatures for different subtypes of polyps based on the binding pattern of antibodies or other high affinity recognition molecules.

## Author Contributions

SR has contributed to conceptualizing, writing, organizing of figures, tables, references, and final revisions. AP has contributed to conceptualizing, literature search, and early drafting of manuscript. MF-C has contributed to conceptualizing, writing, and final revisions. PB has contributed to writing and final revisions. DH has contributed to conceptualizing, writing, final revisions, and supervision of the manuscript. All authors contributed to the article and approved the submitted version.

## Funding

SR and AP acknowledge the UTHealth Innovation for Cancer Prevention Research Training Program (Cancer Prevention and Research Institute of Texas grant #RP160015). PB acknowledges Pancreatic Cancer Action Network (PANCAN; 16-65-BHAT); Cancer Prevention and Research Institute of Texas (CPRIT; RP180873); US National Cancer Institute (NCI; U01 CA214263 and R21 CA185536), Institutional Research Grants and a Startup grant from MD Anderson Cancer Center. 

## Disclaimer

The content is solely the responsibility of the authors and does not necessarily represent the official views of the Cancer Prevention and Research Institute of Texas.

## Conflict of Interest

The authors declare that the research was conducted in the absence of any commercial or financial relationships that could be construed as a potential conflict of interest.
